# Sub-lethal doses of albendazole induce drug metabolizing enzymes and increase albendazole deactivation in *Haemonchus contortus* adults

**DOI:** 10.1186/s13567-020-00820-x

**Published:** 2020-07-23

**Authors:** Pavlína Kellerová, Lucie Raisová Stuchlíková, Petra Matoušková, Karolína Štěrbová, Jiří Lamka, Martina Navrátilová, Ivan Vokřál, Barbora Szotáková, Lenka Skálová

**Affiliations:** 1 Department of Biochemical Sciences, Faculty of Pharmacy, Charles University, Heyrovského 1203, 500 05 Hradec Králové, Czech Republic; 2 Department of Pharmacology and Toxicology, Faculty of Pharmacy, Charles University, Heyrovského 1203, Hradec Králové, Czech Republic

**Keywords:** drug resistance, anthelmintics, benzimidazoles, nematode, UDP-glycosyl transferases, cytochromes P450, P-glycoprotein, ABC-transporters

## Abstract

The efficacy of anthelmintic therapy of farm animals rapidly decreases due to drug resistance development in helminths. In resistant isolates, the increased expression and activity of drug-metabolizing enzymes (DMEs), e.g. cytochromes P450 (CYPs), UDP-glycosyltransferases (UGTs) and P-glycoprotein transporters (P-gps), in comparison to sensitive isolates have been described. However, the mechanisms and circumstances of DMEs induction are not well known. Therefore, the present study was designed to find the changes in expression of CYPs, UGTs and P-gps in adult parasitic nematodes *Haemonchus contortus* exposed to sub-lethal doses of the benzimidazole anthelmintic drug albendazole (ABZ) and its active metabolite ABZ-sulfoxide (ABZSO). In addition, the effect of ABZ at sub-lethal doses on the ability to deactivate ABZ during consequent treatment was studied. The results showed that contact of *H. contortus* adults with sub-lethal doses of ABZ and ABZSO led to a significant induction of several DMEs, particularly *cyp*-*2*, *cyp*-*3*, *cyp*-*6*, *cyp*-*7*, *cyp*-*8*, *UGT10B1*, *UGT24C1*, *UGT26A2*, *UGT365A1*, *UGT366C1*, *UGT368B2*, *UGT367A1*, *UGT371A1*, *UGT372A1* and *pgp*-*3*, *pgp*-*9.1*, *pgp*-*9.2*, *pgp*-*10.* This induction led to increased formation of ABZ metabolites (especially glycosides) and their increased export from the helminths’ body into the medium. The present study demonstrates for the first time that contact of *H. contortus* with sub-lethal doses of ABZ (e.g. during underdose treatment) improves the ability of *H. contortus* adults to deactivate ABZ in consequent therapy.

## Introduction

Haemonchosis is one of the most important parasitic diseases of small ruminants. The nematode *Haemonchus contortus* (*H. contortus*), blood-feeding parasite in abomasum, has a broad range of host species, frequent and cosmopolitan geographic distribution, as well as a significant impact on livestock production. When the environmental conditions support the free-living larval stages, a heavy burden of adult helminths may occur in the hosts, leading to severe anemia which may result in death of animals [1, 2]. Appropriate pasture management should be a primary way to control haemonchosis, but this type of prevention is not sufficient in many cases. In spite of the advances in vaccination development [3], anthelmintic drugs are still the most important tool in the prophylaxis and treatment of *H. contortus* infection in grazing sheep [4]. The efficacy of anthelmintic therapy, however, decreases due to drug-resistance development in helminths, with the helminths’ high fertility and short generational interval providing an enviable developmental plasticity in terms of adaptation and the fast development of drug resistance in *H. contortus* [1]. Among the many mechanisms of drug resistance, some are based on the increased expression and activity of drug-metabolizing enzymes (DME) [5, 6]. These proteins protect all organisms against the potential negative action of drugs and other xenobiotics, which can be metabolized (in series or independently) by three processes, termed Phase I to Phase III. In brief, Phase I enzymes add or uncover a polar group (e.g. hydroxyl group, amino group), while in Phase II a xenobiotic and/or its metabolite is conjugated with an endogenous component such as glucose or glutathione in order to reduce reactivity and usually to increase solubility. In Phase III, xenobiotics and/or their metabolites are transported across membranes to facilitate their biotransformation and elimination [6].

Nematodes possess a relatively large number of DME genes, much higher than other helminth classes [6]. Owing to this fact, nematodes are able to effectively metabolize many anthelmintics and form various types of anthelmintic metabolites [7, 8]. Moreover, drug-resistant nematodes have been shown to form higher amounts of inactive metabolites than did drug-sensitive nematodes [8–10]. It has become clearer that DMEs play an important role in drug resistance in nematodes. From this point of view, three DME super-families attract particular attention: cytochromes P450 (CYPs), UDP-glycosyltransferases (UGTs) and ATP-binding cassette (ABC) transporters. CYPs are the main enzymes catalyzing Phase I of drug metabolism, particularly drug oxidation. In *H. contortus*, constitutive expression is highest in the larval stages for the majority of CYPs, although the pronounced expression of many CYPs was also detected in the adults, especially in the helminth intestine [11].

Thirty-two isoforms of UGTs, the most important Phase II enzymes catalyzing conjugation of UDP-activated sugar donors to small lipophilic chemicals, were identified in *H. contortus* genome. Constitutive expression of isoform UGT368B2 was significantly higher in *H. contortus* resistant strains than in the sensitive strains [12].

ABC transporters are implicated in the transport of a variety of drugs and other xenobiotics across cellular membranes. The participation of several ABC transporters in drug resistance, particularly P-glycoproteins (P-gps), have been described in many parasites [13]. A recent study evaluated the increased expression of P-gp genes in a highly-resistant isolate of *H. contortus* [14].

Although many studies, including the studies mentioned above, have demonstrated the increased expression of some DMEs in resistant isolates in comparison to sensitive ones, many questions remain unanswered. For example, the mechanisms and circumstances under which this increase in expression occurs is not well known. We hypothesize that the induction of certain DMEs in *H. contortus* via contact with sub-lethal doses of anthelmintics which occurs during inaccurate treatment might play important role. Therefore, the present ex vivo study was designed to evaluate the effect of the benzimidazole anthelmintic drug albendazole (ABZ) and its active metabolite ABZ-sulfoxide (ABZSO, the main compound in plasma of ABZ-treated animals) at sub-lethal doses on the expression of CYPs, UGTs and P-gps in adults of *H. contortus*. In addition, the effect of ABZ at sub-lethal doses on the ability to deactivate ABZ during consequent exposure was studied with the aim of determining not only the transcriptional response but also the functional response to anthelmintics.

## Materials and methods

### Parasites

In this ex vivo study we used a susceptible isolate of *H. contortus* ISE: the inbred susceptible-Edinburgh strain (MHco3) [15]. Five lambs 3–4 months old and free of parasites were orally infected with 6000 third stage larvae (L3) of the *H. contortus* ISE strain. Approximately 7 weeks after infection the animals were stunned and exsanguinated in agreement with Czech slaughtering rules for farm animals and according to the protocols which have been evaluated and approved by the *Ethics Committee of the Ministry of Education, Youth and Sports* (Protocol MSMT-25908/2014-9). The agar method was used to isolate the adult nematodes from the sheep hosts’ abomasum [16]. The time between the death of the animal and the isolation of helminths did not exceed 2 h. The live parasites were washed with phosphate-buffered saline (pH 7.4) (PBS) and manually divided by gender based on morphology.

### The exposure of adults to anthelmintics

Males and females were placed separately into RPMI 1640 media (Sigma-Aldrich, Prague, Czech Republic). For transcriptomic analysis, the adults were split into seven groups (3 sample parallels, 10 females or 15 males per sample to obtain sufficient and comparable amount of RNA or proteins) and incubated in 2 mL RPMI medium with or without the drug at 37 °C in CO_2_/O_2_ incubator (5% CO_2_, at dark). Three groups were exposed to different concentrations of ABZ (0.01 µM, 0.1 µM, 1 µM), three another groups were exposed to different concentrations of ABZSO (0.01 µM, 0.1 µM, 1 µM), and one group was placed into medium only with 0.1% dimethyl sulfoxide (DMSO, solvent) as a control. All groups exposed to ABZ were subsequently exposed to 0.1% DMSO. A stock solution of 1 mM ABZ and ABZSO (Sigma-Aldrich, Prague, Czech Republic) was prepared in DMSO followed by serial dilutions in DMSO to produce 3 separate stock concentrations (10 µM, 100 µM and 1000 µM). The incubations lasted 4 and 12 h. In all the groups, the nematodes were alive (based on motility check) during all incubations regardless of whether ABZ was present or absent in the medium. After 4-h and 12-h exposure, the nematodes were immediately placed into 1 mL TriReagent^®^ (Molecular Research Centre, OH, USA), and stored at − 80 °C for later use.

For functional analysis (summarized in Table 1), the nematodes (males and females separately, 4 sample parallels, 10 females or 15 males per sample) were pre-incubated in 2 mL medium without ABZ (control group) or with ABZ at 0.01, 0.1 and 1.0 µM concentrations (affected groups) for 18 h. After pre-incubations, the nematodes were manually washed in PBS twice and placed in fresh medium for incubation. First parallels (blanks) of each group were placed into the medium without ABZ, and second, third and fourth parallels into the medium with 10 µM ABZ (the standard concentration of ABZ for metabolism studies which does not kill nematodes [8, 10, 17]) and incubated for 12 h. After incubation, the nematodes were washed twice in PBS, with both the nematodes and medium separately inserted in microtubes and stored in a freezer (− 20 °C). This experiment was repeated twice.

**Table 1:** **Experimental design of functional analysis**

	Pre-incubation 18 h					Incubation 12 h	
	Control	Affected				Control	Affected
Blanks	DMSO 0.1%	0.01 µM ABZ	0.1 µM ABZ	1.0 µM ABZ	WASHING	DMSO 0.1%	DMSO 0.1%
1	DMSO 0.1%	0.01 µM ABZ	0.1 µM ABZ	1.0 µM ABZ		10 µM ABZ	10 µM ABZ
2	DMSO 0.1%	0.01 µM ABZ	0.1 µM ABZ	1.0 µM ABZ		10 µM ABZ	10 µM ABZ
3	DMSO 0.1%	0.01 µM ABZ	0.1 µM ABZ	1.0 µM ABZ		10 µM ABZ	10 µM ABZ

Affected groups of helminths were pre-incubated with sub-lethal concentrations of ABZ (0.01, 0.1 and 1.0 µM) for 18 h. Control groups were pre-incubated with no ABZ. Both affected and control groups were followingly incubated with high ABZ concentration (10 µM) for 12 h except the blanks.

### RNA extraction and cDNA synthesis

Total RNA was extracted using TriReagent^®^ according to the manufacturer’s protocol following the homogenization of the samples in the FastPrep-24 5G Homogenizer (MP Biomedicals, France). RNA concentrations and purity were determined spectrophotometrically using the NanoDrop ND-1000 UV–Vis Spectrophotometer (Thermo Fisher Scientific, MA, USA) at a wavelength of 260 and 280 nm. The samples were analyzed by the Agilent 2100 Bioanalyzer on RNA Nano chips (Agilent Technologies, CA, USA). Four µg of RNA were treated with DNase I (NEB, UK) and diluted to a concentration of 0.1 µg/µL. One half microgram of the total RNA, random hexamers and Protoscript^®^ II Reverse Transcriptase (NEB, UK) were used for reverse transcription (in 20 µL reaction mixture) according to the manufacturer’s protocol, following which the obtained cDNA was diluted 10× and stored at − 20 °C.

### Quantitative PCR (qPCR)

The changes in expression of the selected CYPs, UGTs and P-gps in *H. contortus* were analyzed by qPCR analyses performed in the 384-Well PCR Thermal Cycler; QuantStudio™ 6 Flex Real-Time PCR System (Applied Biosystems, CA, USA) with SYBR Green I detection. Approximately 5 ng of cDNA was added into a reaction mixture consisting of qPCR Xceed SG 1-step 2× Mix Lo-ROX (IAB, Czech Republic), both forward and reverse primers (final concentration 100 nM), in a final volume of 6 µL. The PCR cycling conditions were initiated by a denaturation step of 2 min at 95 °C, followed by 40 cycles of two step amplification as follows: denaturation for 15 s at 95 °C, annealing for 30 s at 60 °C. Fluorescence data were acquired during the last step. A dissociation protocol with a gradient (0.5 °C every 30 s) from 65 to 95 °C was used to investigate the specificity of the qPCR reaction and presence of primer dimers. Gene-specific amplification was confirmed by a single peak in the melting curve analysis. Samples were run in three biological and two technical replicates, with relative expression calculated based on the “Delta–Delta Ct method” [18]. Two housekeeping genes, glyceraldehyde-3P-dehydrogenase (gpd) and nuclear-cap binding protein subunit 2-like (ncbp), were used as reference genes for the qPCR assay [19]. The primer sequences were either used as previously published or newly designed in Primer3 software [20] with predicted melting temperature 60 ± 2 °C, lengths of 20-23 nucleotides (nt) and GC contents of > 45%. Each gene was checked by Mfold at 60 °C [21] to avoid the region of hairpin structure. All primer sequences were synthesized by Generi Biotech, Czech Republic. The specificity and efficiency of the primers were checked for the qPCR conditions used. The primer sequences, amplicon sizes, and efficiencies are listed in Additional file 1.

### Analysis of ABZ and its metabolites

The adult nematodes (female and male separately) were homogenized repeatedly six-times for 30 s in cooled 0.1 M phosphate buffer (pH 7.4) using the FastPrep homogenizer, after which the homogenates were centrifuged at 3000 × *g* for 5 min. The concentration of protein in the homogenates of the nematodes was measured using a bicinchoninic acid assay according to Sigma-Aldrich protocols. Supernatants of the homogenates as well as medium samples were extracted using solid-phase extraction (SPE) as described previously [22]. Dry samples were quantitatively reconstituted in a mixture of acetonitrile/water (30:70, *v/v*) using sonication and a vortex for 5 min.

One microliter of the reconstituted samples was injected into the UHPLC/MS system. UHPLC (Nexera; Shimadzu, Japan) was optimized using a Zorbax RRHD Eclipse Plus 95Å C18 column 150 × 2.1 mm, 1.8 µm (Agilent Technologies, Waldrbronn, Germany) at a temperature of 40 °C, flow rate 0.4 mL/min and injection volume 1 μL. The mobile phase consisted of water (A) and acetonitrile (B), both with the addition of 0.1% formic acid (MS grade). The linear gradient was as follows: 0 min—15% B, 8 min—40% B, 10 min—95% B followed by 1 min of isocratic elution. The QqQ mass spectrometer (LC–MS-8030 triple quadrupole mass analyzer; Shimadzu, Japan) was used with the following setting of tuning parameters: capillary voltage 4.5 kV, heat block temperature 400 °C, DL line temperature 250 °C, the flow rate and pressure of nitrogen were 12L/min, respectively. ESI mass spectra were recorded in the range of *m/z* 50–1000 in the positive-ion mode, a greater sensitivity for the studied metabolites. The detected metabolites were identified based on the presence of the protonated molecules [M+H]^+^. The isolation width ∆*m/z* 2 and the collision energy 25 eV (determined as the optimal energy for fragmentation of the studied metabolite ions) were used. Argon was the collision gas for MS/MS experiments. The standards of the potential metabolites were generally not commercially available, and they were not prepared due to the difficulties involved in their synthesis. For this reason, the amounts of metabolites were semi-quantified using a ratio of peak areas for the metabolites, with the area of the internal standard (IS) peak (mebendazole). In the homogenates of the nematodes, these ratios were normalized to a milligram of total protein. All data are presented as arithmetic mean ± S.D. (n = 4).

### Statistical analysis

The data for statistical analysis were expressed as the mean ± S.D. (3 biological replicates of each sample for gene expression and 4 biological replicates for functional analysis, each replicate consisted of 10 females or 15 males). Gene expression differences between the treatment groups and incubation times were determined using a two-way ANOVA with Dunnett’s multiple comparisons test. Statistical comparisons of the ABZ metabolites were carried out using the Student’s t-test. All statistical tests were performed in GraphPad Prism^®^ software 8.0.1 (GraphPad Prism, USA), with differences considered significant at P ˂ 0.05.

## Results

### Transcriptional response of CYPs to exposure to sub-lethal concentrations of ABZ and ABZSO in *H.* *contortus* adults

**Figure 1:**
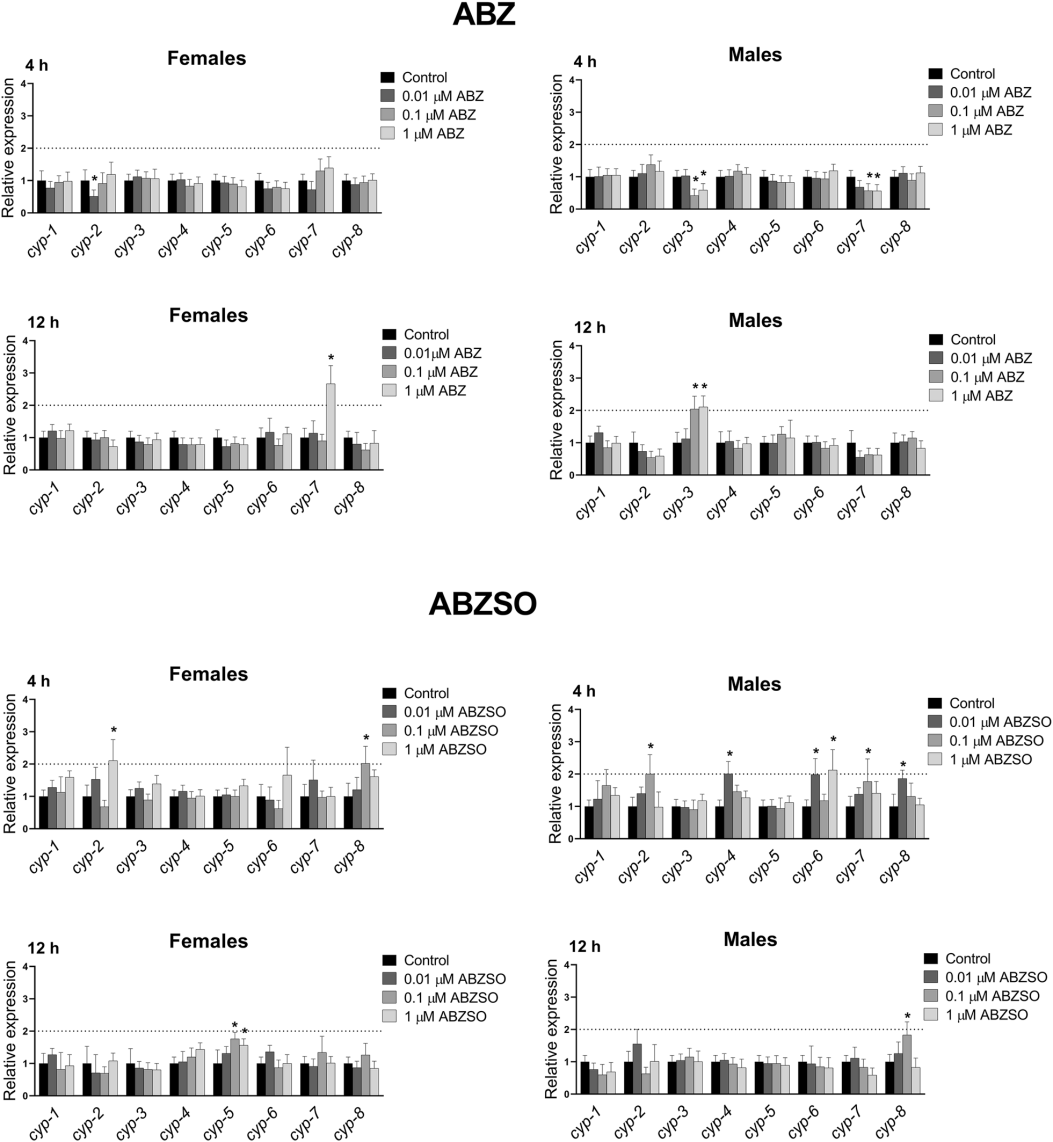
**The comparison of expression levels of cytochromes P450 (CYPs) mRNA in male and female adult*H. contortus*ISE isolates in response to sub-lethal ABZ and ABZSO concentrations.** Adults were exposed to ABZ and ABZSO (0.01 µM, 0.1 µM, 1 µM) for 4 and 12 h. Expression of the genes was analyzed using the housekeeping genes glyceraldehyde-3P-dehydrogenase (gpd) and the nuclear-cap binding protein subunit 2-like (ncbp) as reference genes. Data represent the mean ± S.D. (n = 3). *P < 0.05.

The expression levels of 8 individual CYPs (Wormbase ID numbers are listed in Additional file 1: Table S1) in control nematodes (females and males separately) and in nematodes exposed to sub-lethal concentrations of ABZ and ABZSO for 4 and 12 h were analyzed and compared. The exposures of *H. contortus* adults to sub-lethal ABZ concentrations significantly changed the mRNA expression of only three of the studied CYPs (Figure 1). *Cyp*-*2* in females, *cyp*-*3* and *cyp*-*7* in males were significantly decreased after 4-h ABZ exposure. However, c*yp*-*7* in females (exposed to 1 μM ABZ) and *cyp*-*3* in males (exposed to 0.1 and 1 μM ABZ) were up-regulated twice as much compared to control after 12-h ABZ exposure (at 0.1 µM and 1 µM concentrations). Sub-lethal doses of ABZSO affected expression of more CYPs genes then did ABZ. Four-hour exposure to ABZSO significantly increased expression of *cyp*-*2* and *cyp*-*8* in both genders, and *cyp*-*4*, *cyp*-*6*, *cyp*-*7* and *cyp*-*8* in males. However, only 2 genes, *cyp*-*5* in females and *cyp*-*8* in males, were up-regulated after 12-h ABZSO exposure. The induction effect was mild and only at one concentration and one-incubation time in most cases. In females, 1 µM ABZSO induced *cyp*-*2* (4-h exposure) and cyp-5 (12-h exposure), while 0.1 µM ABZSO induced *cyp*-*8* (4-h exposure) and *cyp*-*5* (12-h exposure). In males, 4-h exposure increased expression of *cyp*-*1*, *cyp*-*2*, *cyp*-*7* (all genes at 0.1 µM ABZSO), *cyp*-*4*, *cyp*-*8* (both genes at 0.01 µM ABZSO) and *cyp*-*6* (at 0.01 and 1 µM ABZSO).

### Transcriptional response of UGTs to exposure to sub-lethal concentrations of ABZ and ABZSO in *H. contortus* adults

**Figure 2:**
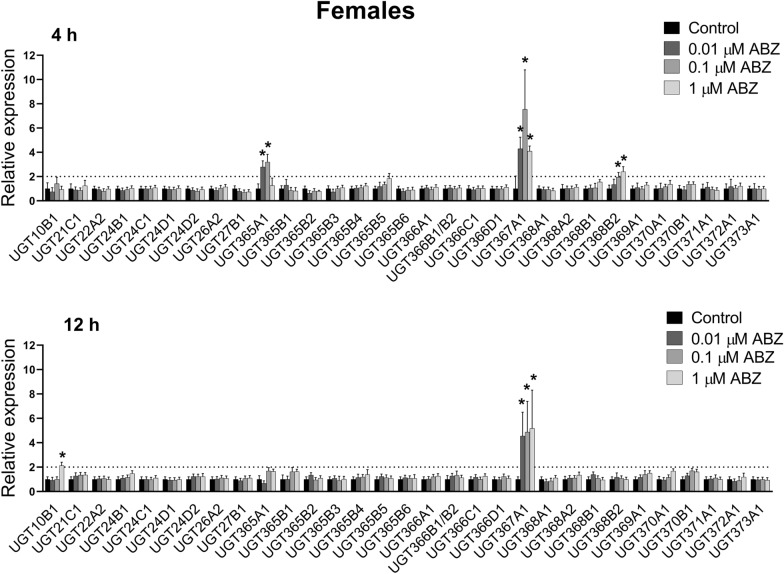
**The comparison of expression levels of UDP-glycosyltransferases (UGTs) mRNA in female adult*H. contortus*ISE isolates in response to sub-lethal ABZ at 0.01** **µM, 0.1** **µM, 1** **µM concentration for 4 and 12** **h.** Expression of the gene was analyzed using the housekeeping genes glyceraldehyde-3P-dehydrogenase (gpd) and the nuclear-cap binding protein subunit 2-like (ncbp) as reference genes. Data represent the mean ± S.D. (n = 3). *P < 0.05.

**Figure 3:**
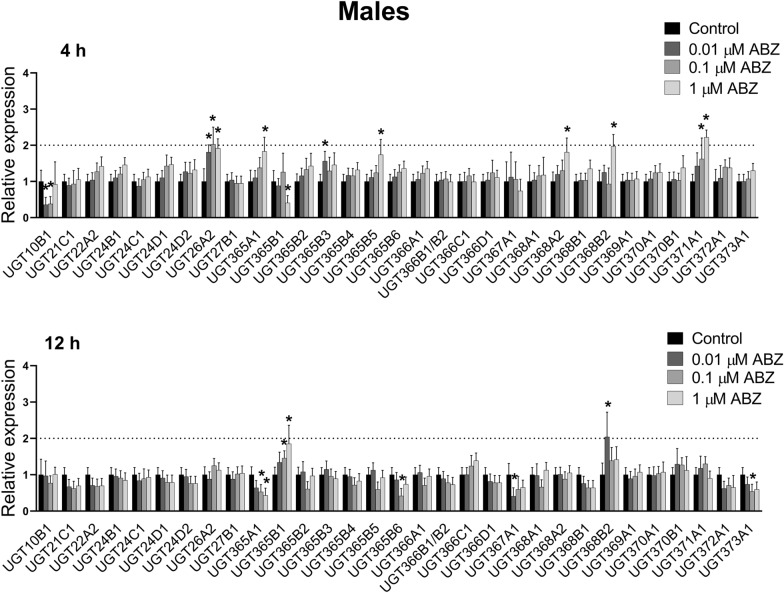
**The comparison of expression levels of UDP-glycosyltransferases (UGTs) mRNA in male adult*H. contortus*ISE isolates in response to sub-lethal ABZ at 0.01** **µM, 0.1** **µM, 1** **µM concentration for 4 and 12** **h.** Expression of the gene was analyzed using the housekeeping genes glyceraldehyde-3P-dehydrogenase (gpd) and the nuclear-cap binding protein subunit 2-like (ncbp) as reference genes. Data represent the mean ± S.D. (n = 3). *P < 0.05.

The expression of UGT genes in control *H. contortus* adults and in adults exposed to sub-lethal concentrations of ABZ and ABZSO for 4 and 12 h were analyzed. After 4-hour ABZ exposure, expression of *UGT365A1*, *UGT367A1* and *UGT368B2* was elevated in females (Figure 2). *UGT10B1* and *UGT367A1* showed increased expression in females exposed to ABZ for 12-h. Interestingly, ABZ at all three concentrations and both time intervals showed substantially and highly elevated *UGT367A1* expression in the females (> 4-times). In the males, expression of twelve UGT genes were changed after ABZ exposure (Figure 3). Seven genes were up-regulated after 4-h exposure: *UGT26A2*, *UGT365A1*, *UGT365B3*, *UGT365B5*, *UGT368A2*, *UGT368B2*, *UGT371A1*, and two genes after 12-h exposure: *UGT365B1* and UGT368B2. Transcription levels of six genes were mildly decreased: *UGT10B1* and *UGT365B1* after 4-h exposure, and *UGT365A1*, *UGT365B6*, *UGT367A1* and *UGT373A1* after 12-h exposure. In males, UGT26A2 seems to be the most interesting, as ABZ at all concentrations increased its expression.

**Figure 4:**
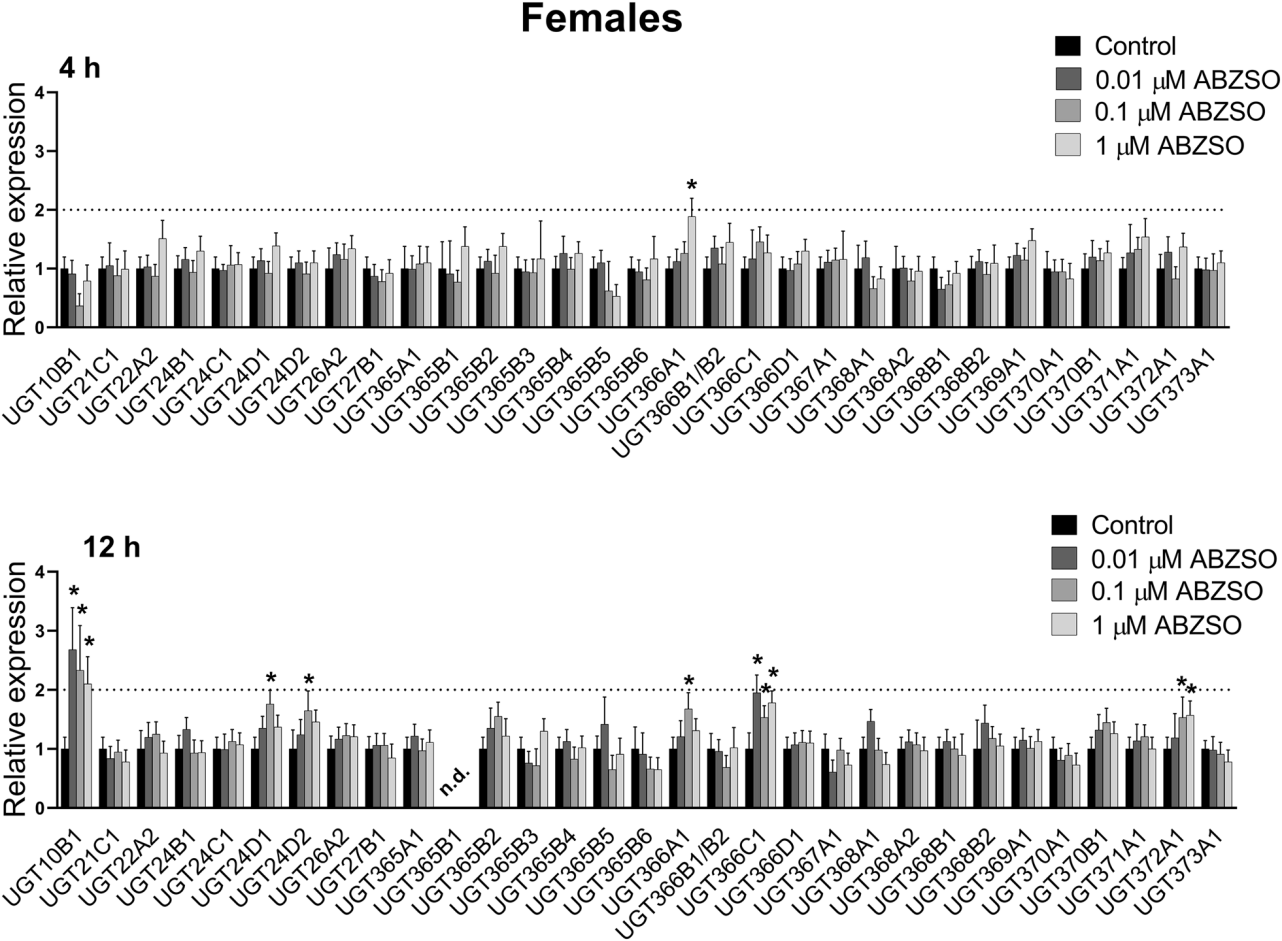
**The comparison of expression levels of UDP-glycosyltransferases (UGTs) mRNA in female adult*H. contortus ISE*isolates in response to sub-lethal ABZSO at 0.01** **µM, 0.1** **µM, 1** **µM concentration for 4 and 12** **h.** Expression of the gene was analyzed using the housekeeping genes glyceraldehyde-3P-dehydrogenase (gpd) and the nuclear-cap binding protein subunit 2-like (ncbp) as reference genes. Data represent the mean ± S.D. (n = 3). *P < 0.05; n.d. = not detected.

**Figure 5:**
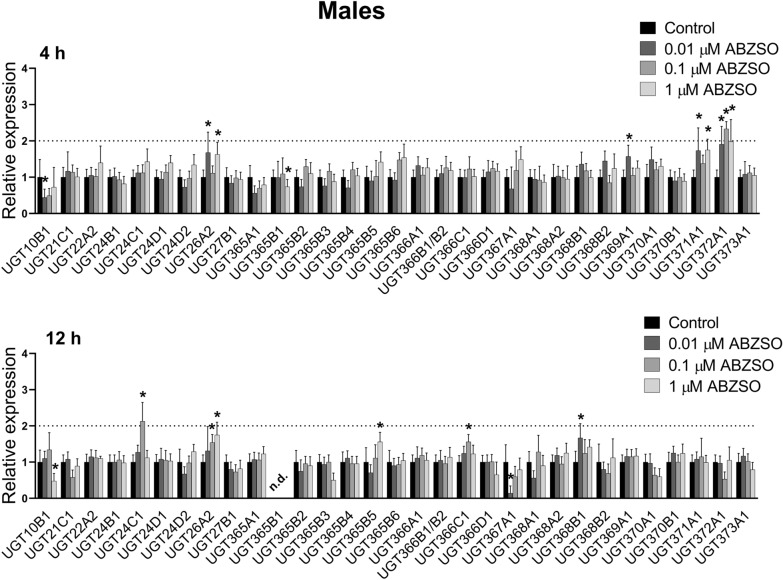
**The comparison of expression levels of UDP-glycosyltransferases (UGTs) mRNA in male adult*H. contortus*ISE isolates in response to sub-lethal ABZSO at 0.01** **µM, 0.1** **µM, 1** **µM concentration for 4 and 12** **h.** Expression of the gene was analyzed using the housekeeping genes glyceraldehyde-3P-dehydrogenase (gpd) and the nuclear-cap binding protein subunit 2-like (ncbp) as reference genes. Data represent the mean ± S.D. (n = 3). *P < 0.05; n.d. = not detected.

In the females, ABZSO exposure induced *UGT366A1* expression after both 4- and 12-h exposures (Figure 4). Whereas after 4-h exposure no other UGT gene had changed expression, increased expressions of 5 other UGT genes (*UGT10B1*, *UGT24D1*, *UGT24D2*, *UGT366C1*, *UGT372A1*) were observed after 12-h exposure. Induction of *UGT10B1* was the most pronounced, occurring at all ABZSO concentrations. In the males, ABZSO-induced overexpression of four UGTs was detected, with *UGT372A1* being the most elevated after 4-h exposure (Figure 5). Expressions of five UGTs were up-regulated and two down-regulated after 12-h ABZSO exposure.

### Transcriptional response of P-gps to exposure to sub-lethal concentrations of ABZ and ABZSO in *H. contortus* adults

**Figure 6:**
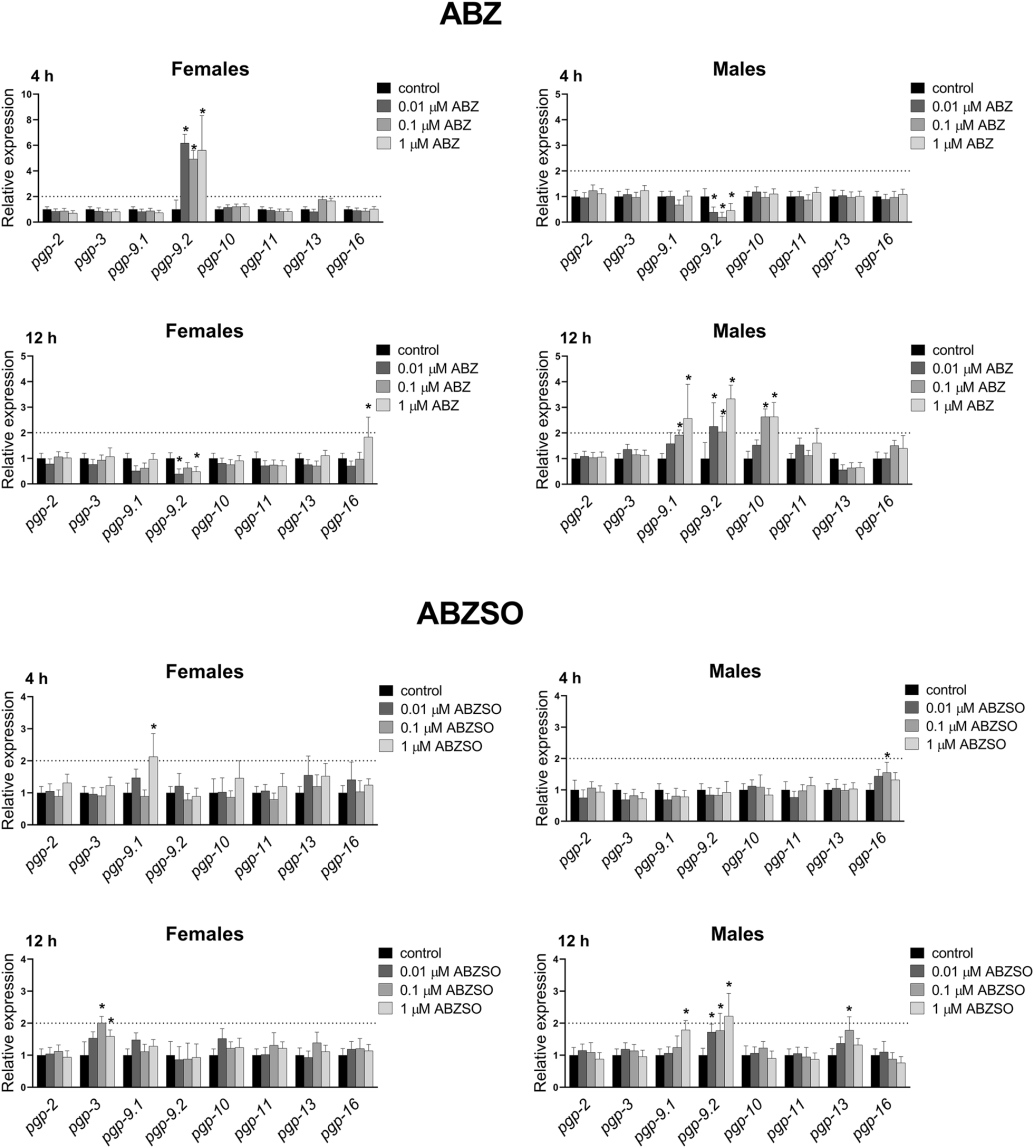
**The comparison of expression levels of P-glycoproteins (P-gps) mRNA in female and male adult*H. contortus*ISE isolates in response to sub-lethal ABZ and ABZSO at 0.01** **µM, 0.1** **µM, 1** **µM concentration for 4 and 12** **h.** Expression of the gene was analyzed using the housekeeping genes glyceraldehyde-3P-dehydrogenase (gpd) and the nuclear-cap binding protein subunit 2-like (ncbp) as reference genes. Data represent the mean ± S.D. (n = 3). *P < 0.05.

In our ex vivo study, exposure to ABZ and ABZSO affected transcription levels of six (out of eight tested) transporter genes in *H. contortus* adults (Figure 6). Concerning ABZ, a 4-h exposure of the females to all three concentrations resulted in significant up-regulation of *pgp*-*9.2* expression in comparison to controls, but this increase dropped after 12-h exposure significantly. However, a 4-h ABZ exposure in the males caused *pgp*-*9.2* down-regulation, while 12-h ABZ exposure (all three concentrations) led to significant *pgp*-*9.2* up-regulation. The other genes with induced levels after 12-h ABZ exposure were *pgp*-*9.1*, *pgp*-*10* in the males and *pgp*-*16* in the females. Concerning ABZSO, 4-h exposure increased *pgp*-*9.1* in the females and *pgp*-*16* in the males. The 12-h ABZSO exposure slightly increased *pgp*-*3* in the females, and *pgp*-*9.1*, *pgp*-*9.2* (both after 1 µM ABZSO) and *pgp*-*13* (only after 0.1 µM ABZSO) in the males.

### Effect of ABZ at sub-lethal doses on *H. contortus* ability to metabolize ABZ

The *H. contortus* adults were pre-incubated in medium without ABZ (control group) and with ABZ at 0.01, 0.1 and 1.0 µM concentrations (affected groups) for 18 h. The *H. contortus* adults were then placed in fresh medium without ABZ (blanks) or with 10 µM ABZ and incubated for 12 h. Qualitative and semi-quantitative analyses of ABZ metabolites in homogenate from the nematodes and in medium was performed using UHPLC/MS. The metabolites were identified based on their fragmentation ions as described previously [10]. The designation and description of the metabolites is presented in Table 2. ABZ metabolites were formed via *S*-oxidation or hydroxylation, hydrolysis and glycosidation followed by *O*-acetylation.

**Table 2:** **Biotransformation of ABZ in*H. contortus*adults—the metabolites detected by UHPLC-MS/MS**

t_R_ (min)	Theoretical *m/z* values of [M+H]^+^ ions	Elemental composition	Description of metabolite formation		Product ions of [M+H]+, *m/z*	Metabolite designation
			Phase I	Phase II		
2.0	444.14	C_18_H_25_N_3_O_8_S	*S*-oxidation	*N*-glycosidation	282, 240, 208	M1_ABZ_
2.5	444.14	C_18_H_25_N_3_O_8_S	*S*-oxidation	*N*-glycosidation	282, 240, 208	M2_ABZ_
3.4	282.09	C_12_H_15_N_3_O_3_S	*S*-oxidation	–	240, 208, 191,159	M3_ABZ_
4.3	370.14	C_16_H_23_N_3_O_5_S	Hydrolysis	*N*-glycosidation	208	M4_ABZ_
5.2	298.09	C_12_H_15_N_3_O_4_S	2*S-oxidation	–	266, 224, 159	M6_ABZ_
5.1	428.15	C_18_H_25_N_3_O_7_S	–	*N*-glycosidation	266, 234	M7_ABZ_
5.1	428.15	C_18_H_25_N_3_O_7_S	+*O*, hydrolysis	Glycosidation, *O*-acetylation	208	M11_ABZ_
5.3	428.15	C_18_H_25_N_3_O_7_S	+*O*, hydrolysis	Glycosidation, *O*-acetylation	208	M12_ABZ_
5.6	428.15	C_18_H_25_N_3_O_7_S	+*O*, hydrolysis	Glycosidation, *O*-acetylation	208	M13_ABZ_
6.1	428.15	C_18_H_25_N_3_O_7_S	+*O*, hydrolysis	Glycosidation, *O*-acetylation	208	M10_ABZ_
6.1	428.15	C_18_H_25_N_3_O_7_S	–	*N*-glycosidation	266, 234	M8_ABZ_
6.7	428.15	C_18_H_25_N_3_O_7_S	–	*N*-glycosidation	266, 234	M9_ABZ_
7.4	266.10	C_12_H_15_N_3_O_2_S	–	–	234	ABZ (parent drug)

**Figure 7:**
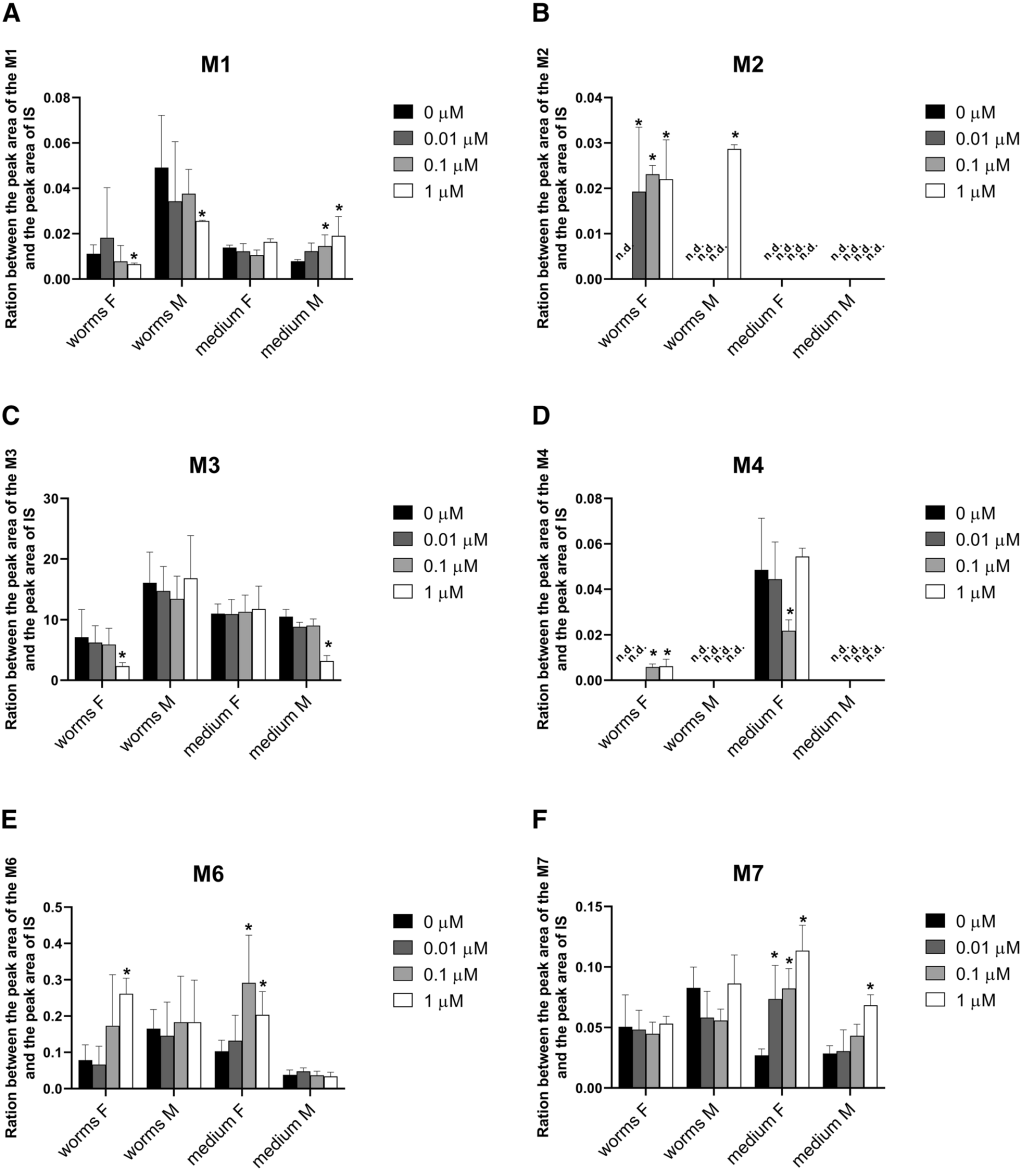

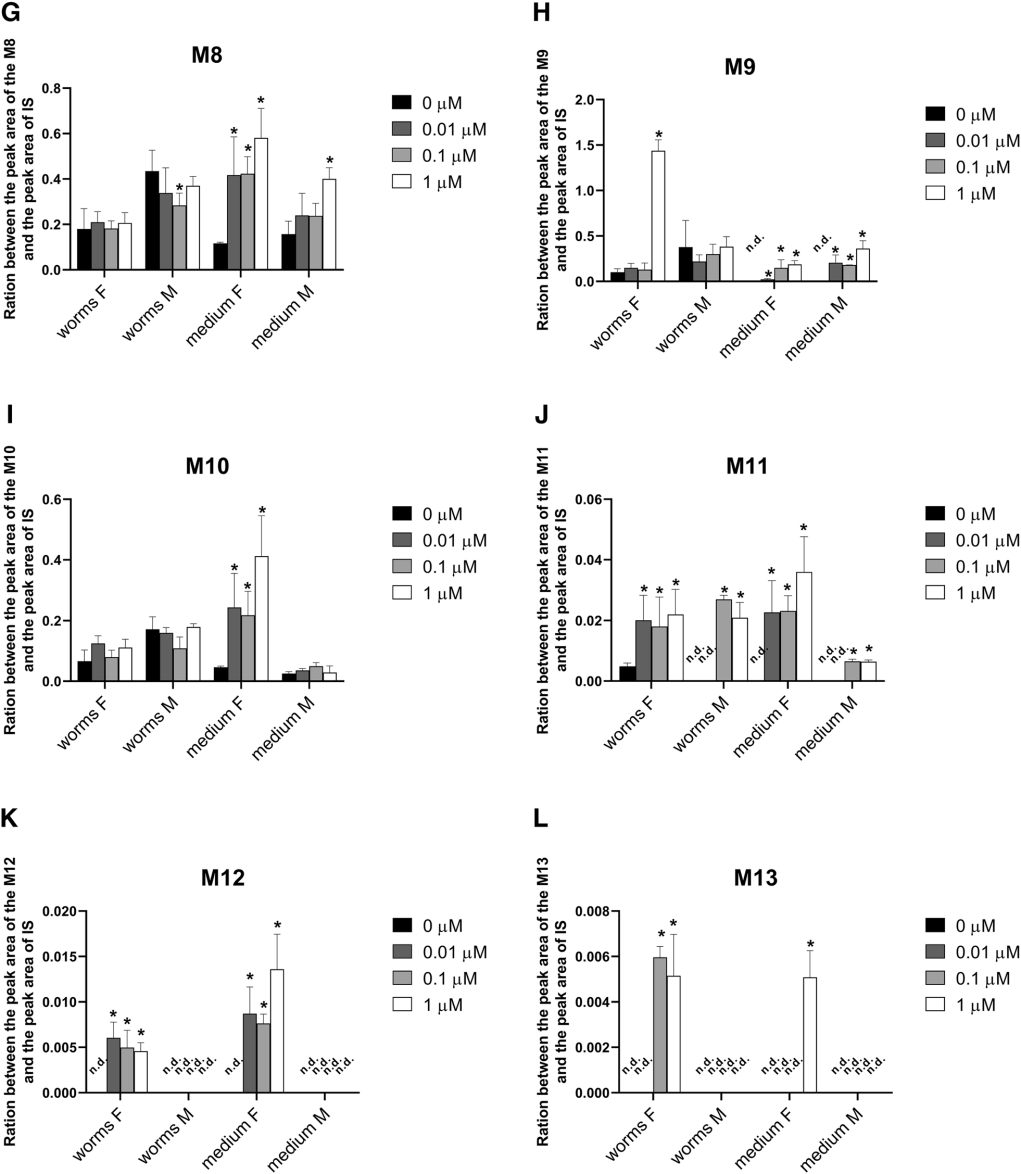
**The comparison of the amounts of individual metabolites presented in homogenate and cultivation medium of male (M) and female (F) of*H. contortus*adults after 12** **h incubation in 10** **µM ABZ concentration with prior 18** **h pre-incubation with ABZ at 0.01, 0.1 and 1.0** **µM concentrations or without.** Individual metabolites: M1 and M2 (*N*-glycoside of ABZSO; **A**, **B**, M3 (ABZSO; **C**), M4 (*N*-glycoside of hydrolyzed ABZ; **D**), M6 (ABZ-sulfone; **E**), M7, M8 and M9 (*N*-glycoside of ABZ; **F**–**H**), M10–M13 (acetylglycoside of ABZ; **I**–**L**). The data represent the mean ± S.D. (n = 3). An asterisk (*) indicates a significant difference (P < 0.05) compared to pre-incubation without ABZ concentration. n.d. = not detected.

**Figure 8:**
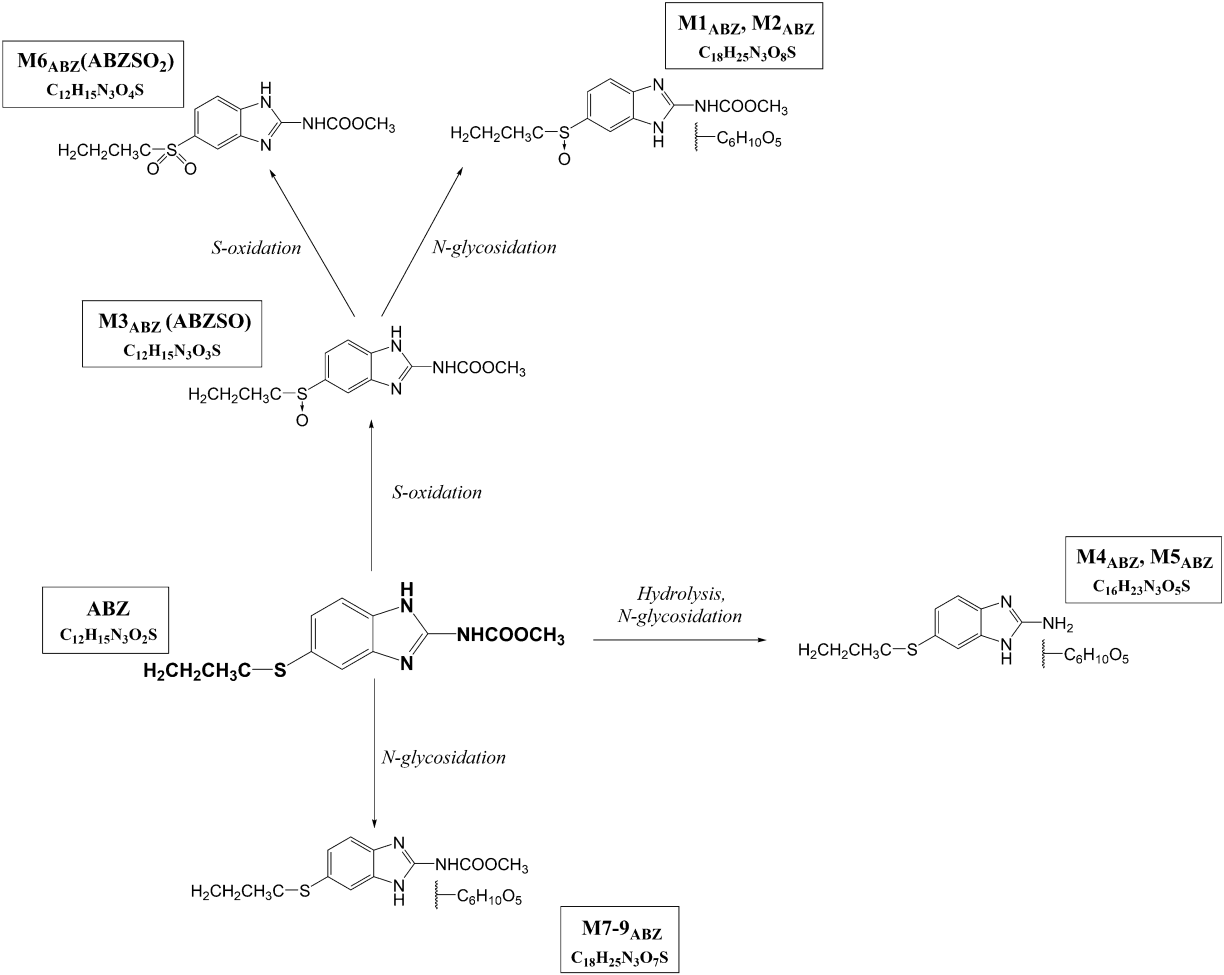
**The proposed metabolic pathway of ABZ in*H. contortus*adults**

The presence/absence of individual metabolites in the control samples (homogenate and medium from the nematodes pre-incubated in medium without ABZ), and the affected samples (homogenate and medium from the nematodes pre-incubated with 0.01, 0.1 and 1.0 µM ABZ) is summarized in Table 3. Semi-quantification allowed the comparison of the amounts of individual metabolites present in the homogenate and cultivation medium of male and female nematodes with or without pre-incubation, with the results shown in Figure 7. The scheme of metabolic pathway of ABZ in *H. contortus* adults is presented in Figure 8. In the medium with the males, the amount of the metabolite M1 (*N*-glycoside of ABZSO) rose with increasing ABZ concentrations in pre-incubation. On the other hand, the M1 formation in females was not affected by ABZ (Figure 7A).

**Table 3:** **Presence (+) or absence (-) of individual ABZ metabolites (M1–M13) in homogenates and medium of*H. contortus adults*preincubated without (0) or with ABZ in sub-lethal doses (0.01, 0.1 and 1** **µM)**

Metabolite designation	Homogenate of *H. contortus*								Medium							
	Female				Male				Female				Male			
	0	0.01	0.1	1	0	0.01	0.1	1	0	0.01	0.1	1	0	0.01	0.1	1
M1_ABZ_	+	+	+	+	+	+	+	+	+	+	+	+	+	+	+	+
M2_ABZ_	−	+	+	+	−	−	−	+	−	−	−	−	−	−	−	−
M3_ABZ_	+	+	+	+	+	+	+	+	+	+	+	+	+	+	+	+
M4_ABZ_	−	−	+	+	−	−	−	−	+	+	+	+	−	−	+	+
M6_ABZ_	+	+	+	+	+	+	+	+	+	+	+	+	+	+	+	+
M7_ABZ_	+	+	+	+	+	+	+	+	+	+	+	+	+	+	+	+
M8_ABZ_	+	+	+	+	+	+	+	+	+	+	+	+	+	+	+	+
M9_ABZ_	+	+	+	+	+	+	+	+	−	+	+	+	−	+	+	+
M10_ABZ_	+	+	+	+	+	+	+	+	+	+	+	+	+	+	+	+
M11_ABZ_	+	+	+	+	−	−	+	+	−	+	+	+	−	−	+	+
M12_ABZ_	−	+	+	+	−	−	−	−	−	+	+	+	−	−	−	−
M13_ABZ_	−	−	+	+	−	−	−	−	−	−	−	+	−	−	−	−
ABZ	+	+	+	+	+	+	+	+	+	+	+	+	+	+	+	+

The metabolite M2 (*N*-glycoside of ABZSO) was found only in the *H. contortus* adults pre-incubated with ABZ, and it was not detected in any medium (Figure 7B). ABZSO represents the most abundant ABZ metabolite (M3) in both sexes of *H. contortus* (Figure 7C). Comparing control and ABZ-affected samples, concentrations of M3 did not differ with the exception of a M3 decrease in the female homogenates as well as the male medium pre-incubated with 1 µM ABZ. The metabolite M4 (*N*-glycoside of hydrolyzed ABZ) was formed only in the females. A much higher amount of M4 was detected in the medium than in the helminths (Figure 7D).

The pre-incubation of the *H. contortus* adults with ABZ at low concentrations significantly increased the amount of ABZ-sulfone (M6) in the homogenates and media from the females but not from males (Figure 7E). An increased amount of M7 (*N*-glycoside of ABZ) was found in media from both sexes pre-incubated with ABZ at low concentrations (Figure 7F). A significant increase of the amount of M8 (*N*-glycoside of ABZ) after pre-incubation was observed in medium from males as well as females (Figure 7G). The pre-incubation of females with 1 µM ABZ led to a substantial increase of M9 (*N*-glycoside of ABZ) in the homogenates (Figure 7H). This metabolite was not detected in the control media, but the amounts significantly increased in the medium from the nematodes pre-incubated with ABZ at low concentrations. Pre-incubation significantly induced the formation of the metabolite M10 (acetylglycoside of ABZ) only in the medium from the females, and it increased the amount of the metabolite M11 (acetylglycoside of ABZ) in the males as well as in the females (Figures 7I, J). The minor metabolites M12 and M13 (acetylglycosides of ABZ) were female-specific, and they were detected only in the helminths pre-incubated with ABZ at low concentrations (Figures 7K, L).

## Discussion

Contact of helminths with sub-lethal doses of anthelmintics may occur relatively often and under various circumstances, with the improper management of anthelmintic administration such as underdosage representing the main cause [23, 24]. The appropriate dosage of drugs is an issue for most large animal farms. Even though that the treatment dosage should be adjusted to the heaviest animal from the flock [25], most farmers do not weigh all the animals, and therefore some of them are easily undertreated. Moreover, in some regions the drug is mixed into food, in which case many animals may not uptake a sufficient amount of the drug. In addition to dose, the available concentration of anthelmintic basically depends on drug absorption, distribution, metabolism and elimination, any of which might vary due to several factors e.g. animal species, breed, age, weight, sex and nutrition [26, 27]. These factors along with others are key in terms of the insufficient concentration of the drug affecting the helminths. As several laboratory experiments have shown, sub-lethal doses of anthelmintics contribute to the selection of resistant or tolerant strains of parasites with single-nucleotide polymorphisms (SNPs) at the beta-tubulin 1 gene, which is considered a principal mechanism of ABZ resistance in nematodes [24]. Nevertheless, a number of studies indicate the existence of other mechanisms of ABZ resistance, e.g. a recent study on the nematode *Ancylostoma ceylanicum* describing the preparation of ABZ-resistant strain without these SNPs. [28] The induction of drug-metabolizing enzymes (DMEs) might represent one of these mechanisms.

Although induced expression of certain DMEs in resistant isolates in comparison to sensitive ones has been reported repeatedly [5, 6, 12, 14], the mechanisms and circumstances of this induction have not been elucidated. As the contact of nematodes with sub-lethal doses of anthelmintics may play an important role, we incubated ex vivo *H. contortus* adults from ISE strain (sensitive to all anthelmintics) with the anthelmintic drug albendazole (ABZ) and its active metabolite ABZ-sulfoxide (ABZSO) at three sub-lethal doses for 4 and 12 h. Two different incubation duration were used because it is known that the time required to induce different DMEs differs significantly [29]. The expression levels of individual CYPs, UGTs and P-gps were quantified and compared to levels in controls (exposed to solvent only). We focused on these DMEs, as they have been considered to be key enzymes responsible for ABZ and ABZSO metabolism in *H. contortus* [10].

CYPs are key biotransformation enzymes catalyzing the oxidation of drugs and other xenobiotics in all organisms, with *S*-oxidation representing the main metabolic transformation mechanism of ABZ and ABZSO in *H. contortus* adults. For these reasons, the expression levels of 8 individual CYPs in control adults (females and males separately) and in adults exposed to sub-lethal concentrations of ABZ and ABZSO for 4 and 12 h were analyzed and compared. The exposures of *H. contortus* adults to sub-lethal ABZ concentrations significantly changed the mRNA expression of only three of the studied CYPs, *Cyp*-*2* in females, *cyp*-*3* and *cyp*-*7* in males. Sub-lethal doses of ABZSO affected expression of more CYP genes then did ABZ. However, the induction effect was mild and only at the higher concentration in most cases.

In a previous study of ours, *H. contortus* adults were exposed to sub-lethal doses of ivermectin (IVM). In females, no significant changes in the transcription profile of CYPs were observed except for *cyp*-*7*, which was significantly up-regulated after 12 h exposure time. In the males, three genes were significantly increased: *cyp*-*1*, *cyp*-*3*, and *cyp*-*5* [30]. Comparing the effect of ABZ and ABZSO, IVM had the same effect as ABZ in females (up-regulation only of *cyp*-*7*). Both ABZ and IVM increased transcription of *cyp*-*3* in males. However, ABZSO induced completely different CYP genes than did ABZ and IVM, probably due to higher hydrophilicity. On the other hand, no CYP genes were induced by thiabendazole (2.5 µM) exposure for 3 and 6 h in *H. contortus* larvae [31].

Besides the CYPs, we focused also on UGTs. The association of UGTs with resistance to several drug classes has been shown in human therapy [32, 33]. Recently, UGTs have been considered key players in insecticide resistance [34]. In *H. contortus*, 32 UGTs are present [12], and the formation of glycosides is the main metabolic pathway of ABZ as well as ABZSO in *H. contortus* adults. Moreover, resistant isolates formed many more ABZ- and ABZSO-glycosides than did the susceptible ones [10]. In the present study, we analyzed the expression of UGT genes in control nematodes and in nematodes exposed to sub-lethal concentrations of ABZ and ABZSO for 4 and 12 h. In females, ABZ exposure elevated expression of three and two UGTs after 4 and 12-h, respectively. Interestingly, ABZ at all three concentrations and both time intervals showed substantially and highly elevated *UGT367A1* expression in the females (> 4-fold). In the males, expression of seven UGT genes were upregulated after ABZ exposure. Among them, UGT26A2 seems to be the most interesting, as ABZ at all concentrations increased its expression. Induction of *UGT10B1* in females was the most pronounced, occurring at all ABZSO concentrations. In the males, ABZSO-induced overexpression of four UGTs was detected, with *UGT372A1* being the most elevated after 4-h exposure. Interestingly, the ABZ or ABZSO exposure in our study evaluated two UGTs (*UGT368B2*, *UGT372A1*) in both genders which were also increased in resistant (IRE) and multi-resistant (WR) strains of *H. contortus* for both genders in comparison to the susceptible (ISE) strain [12]. Furthermore, increased constitutive expression of another UGT (*UGT366A1*) in the females as well as three other UGTs (*UGT26A2*, *UGT371A1*, *UGT368B1*) in the males in both resistant strains in comparison to the sensitive strain was reported, suggesting possible contribution to xenobiotics deactivation [12].

In model nematodes *Caenorhabditis elegans*, *ugt*-*22* (*UGT16C1*) was identified as a detoxification enzyme influencing ABZ efficacy. Mutation and overexpression of the *ugt*-*22* gene altered ABZ EC_50_ by 2.3- to 2.5-fold [35]. When *C. elegans* adults of the resistant strain CB3474 ben-1(e1880) III were exposed to ABZ and other benzimidazole anthelmintics (mebendazole, thiabendazole, and oxfendazole), the up-regulation of UGT genes (ugt-14/25/33/34/37/41/8/9) was observed. Surprisingly, the transcriptional xenobiotic response of *H. contortus* adults from the resistant strain UGA/2004 to anthelmintics exposure was undetectable [36]. Nevertheless, this discrepancy is probably based on the very high (lethal) concentrations (1.13 mM) of anthelmintics used in mentioned study.

In addition to CYPs and UGTs, the effect of ABZ and ABZSO on expression of efflux transporters P-glycoproteins (P-gps) was also studied as P-gps have been implicated in the multidrug resistance of different parasitic nematodes in livestock [14]. Although the increased transcription of P-gps has been mainly associated with resistance to IVM and other macrocyclic lactones [37, 38], the participation of P-gps in ABZ resistance cannot be excluded. In our study, exposure to ABZ and ABZSO affected transcription levels of six (out of eight tested) transporter genes in *H. contortus* adults. However, IVM-induced changes in P-gps expression were more pronounced; particularly the expression of *pgp*-*9.2* in the males and *pgp*-*10*, *pgp*-*11* in the females was increased substantially [30], a result which is in agreement with the clear relationship of P-gps with IVM metabolism and resistance in nematodes [39, 40]. Nevertheless, increased transcription of several P-gps after ABZ and ABZSO exposure suggests a particular role of these transporters in the metabolism of these drugs as well.

With the aim of determining whether DMEs induction really improves the ability to deactivate ABZ during consequent exposure, the metabolism of ABZ was compared in control nematodes and in nematodes pre-incubated with a sub-lethal ABZ dose. UHPLC/MS analyses revealed that *H. contortus* adults metabolized ABZ via S-oxidation or hydroxylation, hydrolysis and glycosidation followed by *O*-acetylation. All the metabolites can be considered as deactivation products with exception of ABZSO, which is anthelmintically active (and marketed as ricobendazole). Twelve ABZ metabolites were found in *H. contortus* adults pre-incubated with ABZ, while only nine metabolites were detected in the control population. These nine metabolites were identified and characterized in our previous study of ABZ metabolism in *H. contortus* adults [10]. In our present study, the increased formation of several metabolites (ABZSO_2_ and various glycosides) was found in the adults which were exposed to sub-lethal doses of ABZ. The finding that all the metabolites with increased production can be considered a deactivation product underscores the importance of this process for improving tolerance to ABZ. Moreover, the results of our functional study are in an excellent agreement with our transcriptional study: the expression levels of some CYPs (probably catalyzing ABZ oxidation), several UGTs (catalyzing ABZ and ABZSO glycosidation) and P-gps (exporting ABZ metabolites from helminths’ bodies) were elevated in the nematodes exposed to sub-lethal doses of ABZ. In particular, the up-regulation of UGTs (*UGT10B1*, *UGT365A1*, *UGT367A1*, *UGT368B2*) in combination with the increased formation of nine glycosylated metabolites provides promising results in terms of considering UGTs as anthelmintic resistance mediators. Nevertheless, the evaluation of the role of individual UGTs in ABZ metabolism will require further study. In addition to UGTs, other DMEs in *H. contortus* require further attention, as hydrolases and acetylases also take part in ABZ metabolism, and no information about these enzymes is available.

Even though the parent drug of ABZ is considered neither a substrate nor an inhibitor of P-gps [41], a statistically significant interaction between ABZ metabolites and drug efflux inhibitors were presented in sheep and rats [42]. In our study we clearly showed that both ABZ and ABZSO can induce more than half of the P-gp genes studied. Moreover, a significant increase was shown in the efflux of eight metabolites, a finding which verifies the higher activity of transporters after contact with sub-lethal ABZ concentrations; this likewise suggests that ABZ biotransformation products are better substrates for efflux pumps than ABZ itself. This supports the hypothesis that P-gps do play a significant role for *H. contortus* resistance, not only for ivermectin [43, 44], but probably for other anthelmintic drugs including levamisole, monepantel [45, 46] and also ABZ.

In conclusion, the presented study demonstrates for the first time that contact of *H. contortus* adults with sub-lethal doses of ABZ leads to the induction of several drug-metabolizing enzymes, particularly UGTs, and to a lesser extent CYPs and P-gps. This induction improved the ability of *H. contortus* adults to deactivate ABZ in consequent therapy via the increased formation of inactive metabolites (namely ABZ-sulfone and various glycosides) and their export from the helminths’ body. In this way, the contact of *H. contortus* with sub-lethal doses of ABZ could allow parasites to survive pharmacotherapy.

## Supplementary information

**Additional file 1.** The sequences, amplicon sizes, and efficiencies of primers.

## Data Availability

The datasets supporting the conclusions of this article are included within the article and its additional files.

## Abbreviations

ABZ: albendazole
ABZSO: albendazole sulfoxide
CYP: cytochrome P450
IS: internal standard
ISE: inbred susceptible-Edinburgh strain (MHco3)
P-gp: P-glycoprotein
UGT: UDP-glycosyltransferase
