# Supplementary material for: Sub-lethal doses of albendazole induce drug metabolizing enzymes and increase albendazole deactivation in Haemonchus contortus adults
Source: Vet Res. 2020 Jul 23;51:94. doi: 10.1186/s13567-020-00820-x (PMC7379777; doi:10.1186/s13567-020-00820-x)
Supplement: Supplementary file 1 — Additional file 1. The sequences, amplicon sizes, and efficiencies of primers. [file 13567_2020_820_MOESM1_ESM.docx]

| Table S1 | | | | | | |
| --- | --- | --- | --- | --- | --- | --- |
| Gene ID | WORM BASE ([PRJEB506)](https://parasite.wormbase.org/Haemonchus_contortus_prjeb506/Gene/Summary?g=HCOI01574200) | | Primer Sequence | Product size | Amplification eff. [%] | Source |
|  | Current | Former |  |  |  |  |
| *cyp-1* | HCON_00143950 | HCOI00816200 | F: TGGCAAGTTGAAGAAGGTAGAAG  R: GCCAGTCCTTCTCCTAGACATTG | 75 | 93 | Own design |
| *cyp-2* | HCON_00141020 | HCOI00284400 | F: GTCTCTCCACTTTGATCGCAA  R: CAGAAGATGAGCAAGAACAAGC | 181 | 98 | Own design |
| *cyp-3* | HCON_00022670 | HCOI00383700 | F: TCGGTCAATGTACAGCTGGC  R: CCTGTCATGTATCGGTCCGG | 89 | 92 | Own design |
| *cyp-4* | HCON_00084620 | HCOI02017000 | F: ATCGATCAGTGGGCTTTGCA  R: ACACATTGCCTTGGACCCAA | 140 | 95 | Own design |
| *cyp-5* | HCON_00038960 | HCOI01579500 | F: AGCTGTGCATACTGTCAACGA  R: TCTCCTTCACCGAATTTGTTGT | 93 | 100 | Own design |
| *cyp-6* | HCON_00024010 | HCOI01920700 | F: GGTCTTGCCTTGTTGTTCGA  R: GACAAGATCACCTAGCACTGGA | 102 | 102 | Own design |
| *cyp-7* | HCON_00045430 | HCOI00165400 | F: GCGAATATCCCACTGCCTGA  R: ACCGGAAAACCAAGTGAGGG | 53 | 107 | Own design |
| *cyp-8* | HCON_00022640 | HCOI00383400 | F: TGGAATGTGAATTGGCCGTT  R: CGGAGTTCAGTGGCTTCATC | 64 | 105 | Own design |
| *UGT10B1* | HCON_00104220 | HCOI01037200 | F: TCAACTTGCAATCACCCACG  R: CTGAGAGCGGGATGAGGTAG | 147 | 104 | Own design |
| *UGT21C1* | HCON_00119360 | HCOI01817600 | F: GCCGTGCTGCTGAATTGAAT  R: CGTCTGGATTATCACGAAGAGAG | 69 | 97 | Own design |
| *UGT22A2* | HCON_00135760 | HCOI00058000 | F: GATCTCTGTGGAATCGGCGT  R: TATGAAGGTGGTGCTGGGAC | 119 | 100 | Own design |
| *UGT24B1* | HCON_00126565 | HCOI00244800 | F: ACGTACACCTATTCCAATGGCT  R: CCTTGGTTTTGGGGTGTTGAAG | 60 | 106 | Own design |
| *UGT24C1* | HCON_00122990 | HCOI00868700 | F: GGATCCGAGACATAGGCAGC  R: ACAGTGAGTTTGCATCCCTC | 69 | 93 | Own design |
| *UGT24D1* | HCON_00126560 | HCOI01592800 | F: GAGTAGCAGAGACGTTGGCA  R: TCCCGAAATCATCACCATCGT | 59 | 100 | Own design |
| *UGT24D2* | HCON_00127680 | HCOI00244900 | F: GATCAGTCGCTCCCAGTCAC  R: TGGGTGGCAATCGATCTTTCA | 132 | 90 | Own design |
| *UGT26A2* | HCON_00092310 | HCOI01238200 | F: GGGCCTGGTTGTCTTCTCTC  R: TGTTTCAGTGGGATCGCCTC | 156 | 99 | Own design |
| *UGT27B1* | HCON_00132780 | HCOI01933800 | F: TGTGATGGTTTCGGAATGGA  R: AGCACGGTTTTCTCATCAAGA | 60 | 100 | Own design |
| *UGT365A1* | HCON_00125590 | HCOI01462600 | F: GCTAGCTACGTTCCAGGAGC  R: CAGACGCTCAAAGGTGTTCA | 63 | 92 | Own design |
| *UGT365B1* | HCON_00125530 | HCOI01462400 | F: GCTTGCCAAACACGGTACTG  R: GAATATCCTTCTCAGAGAGTCCC | 139 | 88 | Own design |
| *UGT365B2* | HCON_00125570 | HCOI01461700 | F: ACGACGATGAGCTAATGAAACA  R: ACCGCTGAGAATACACCAATTG | 133 | 100 | Own design |
| *UGT365B3* | HCON_00125540 | HCOI01461900 | F: TCATTCGGTTCAGCAATCAAGG  R: CGAAGACTTCCAGAAAATTCCTCT | 70 | 100 | Own design |
| *UGT365B4* | HCON_00125545 | HCOI01462000 | F: TTCACAAAACCATCCCGCTC  R: CGGCATGTAGATTGATTTAGCCA | 149 | 100 | Own design |
| *UGT365B5* | HCON_00125560 | HCOI01462200 | F: CTTGGTGGAATCGCGGTCTA  R: TTTTGAGGTGCCCATGTGGT | 269 | 100 | Own design |
| *UGT365B6* | HCON_00125550 | HCOI01462100 | F: TCCAAGTTATGTTCCAGGGCAT  R: AGTTCTTCATAATCCTTGAACCGC | 177 | 100 | Own design |
| *UGT366A1* | HCON_00121110 | HCOI02015300 | F: GCAGCCTTTGATGAGCACAC  R: GGCCCACTTGAATGAGACGA | 68 | 100 | Own design |
| *UGT366B1/B2* | HCON_00121182 | HCOI00320100 | F: GGCGATCAAATAAGGAATGCG  R: ATCTGGGATTGTTGAGAACTAGG | 127 | 100 | Own design |
| *UGT366C1* | HCON_00121250 | HCOI01255100 | F: TTATGCCAGCCTCTCTCGGT  R: CACTTGCGATGCCTGTCTG | 111 | 100 | Own design |
| *UGT366D1* | HCON_00121240 | HCOI01255000 | F: CGGGAAGCCATTGATCGAGT  R: TGAAAGGACGTCGCGCTAAT | 88 | 100 | Own design |
| *UGT367A1* | HCON_00165360 | HCOI00538800 | F: AGCGAACTCTGGCCTTCATC  R: TCCGCGAACAATGGTATGGT | 97 | 100 | Own design |
| *UGT368A1* | HCON_00040966 | HCOI01078900 | F: GGAGTTCCCACCAATCCCAG  R: CGTTCACGTTCAGGAGGACA | 229 | 100 | Own design |
| *UGT368A2* | HCON_00040964 | HCOI01452100 | F: TCGGCCAACAGCAAACATTG  R: GTATTGGGGAACGCGGAGAA | 210 | 100 | Own design |
| *UGT368B1* | HCON_00040962 | HCOI00785300 | F: TGGGAAGAAAAATTGAGGCAGTT  R: TGTTTCAGCAGCTTCCTTAAATCA | 74 | 100 | Own design |
| *UGT368B2* | HCON_00040960 | HCOI00785400 | F: CAGTGGGAAAAGGATCTATGGA  R: TGTTTGAGCAATTCCCGCAG | 77 | 100 | Own design |
| *UGT369A1* | HCON_00194230 | HCOI00240800 | F: TCTAGTTTCGTTCCGGCCAC  R: TCGCCCAAGGAACGTCATT | 60 | 100 | Own design |
| *UGT370A1* | HCON_00133660 | HCOI01632300 | F: CCACCGAGAATCTGACAGCC  R: GGGCTGCATTTGAGGTGAATC | 69 | 100 | Own design |
| *UGT370B1* | HCON_00133650 | HCOI01632400 | F: GAGAAACGGATGGATGGCGA  R: ACGGGCTGCATTCGAGTTAA | 133 | 100 | Own design |
| *UGT371A1* | HCON_00108700 | HCOI01917400 | F: CCACACACTCAGCATATCACT  R: GTTCCCTTCGATGTTGGATCA | 160 | 100 | Own design |
| *UGT372A1* | HCON_00161690 | HCOI01985800 | F: CTCGTCGTTTGGGTATCGCT  R: CGAAGCTGGTGTCCGTAAGT | 99 | 100 | Own design |
| *UGT373A1* | HCON_00040490 | HCOI01651100 | F: TCAACCCTCGGAATGATGCC  R: AATGGTGTCAGTCCGGTTGG | 86 | 100 | Own design |
| *pgp-2* | HCON_00004450 | HCOI00025600 | F: GGACAAAAGCAGCGAATTGCC  R: ACAGACGATGCGCTACAATGAC | 169 | 98 | Sarai et al. (2013) |
| *pgp-3* | HCON_00042800 | HCOI00117000 | F: TAGCTTTGGTTGGGCATTCG  R: TCGGAGTAGCAAGCTGATGA | 59 | 95 | Own design |
| *pgp-9.1* | HCON_00130050 | HCOI01671700 | F: TCGACGGGAATCAAGAAATC  R: GCCCATCATTACGGAGAAGA | 168 | 98 | Raza et al. (2016) |
| *pgp-9.2* | HCON_00130060 | HCOI01574200 | F: GACGGGGATGGTGAGGAAG  R: GGATCCGAGGCCCATAAACT | 102 | 99 | Own design |
| *pgp-10* | HCON_00168840 | HCOI00574500 | F: CCGGCAGTAATATGTCCCCT  R: GCAGGAAATTGGAACGAGCA | 89 | 96 | Own design |
| *pgp-11* | HCON_00162780 | HCOI00233200 | F: ACCACGAAGCTGAACGAGAA  R: CACCAGAGTGATACGCCAGTC | 150 | 93 | Raza et al. (2016) |
| *pgp-13* | HCON_00041390 | HCOI01470800 | F: AGACACTATCGCTCAACCATCT  R: GCTCTTGTCCAACTAATGCCATT | 54 | 105 | Own design |
| *pgp-16* | HCON_00035870 | HCOI00062600 | F: AAAAGCGAGACAAGGTCGAA  R: TGTTTGGTTACCATGCTTGC | 165 | 105 | Issouf et al. (2014) |
| *gpd* | CDJ92718.1^*^ |  | F: ACGAGACCTACAATGCAGCC  R: GCGAGACAGTTGGTGGTACA | 67 | 101 | Lecová et al. (2015) |
| *ncbp* | CDJ82645.1^*^ |  | F: CCGAGCAGATACCGAAAATGC  R: CGAAGCCTGCATCATAGTCCA | 89 | 99 | Lecová et al. (2015) |

^*^ NCBI GenBank database
